# Propensity matched analysis examining the effect of passive reversal of direct oral anticoagulants on blood loss and the need for transfusions among traumatic geriatric hip fractures

**DOI:** 10.1186/s40001-023-01053-2

**Published:** 2023-07-21

**Authors:** Richard Meinig, Stephanie Jarvis, Kristin Salottolo, Nnamdi Nwafo, Patrick McNair, Paul Harrison, Steven Morgan, Therese Duane, Bradley Woods, Michelle Nentwig, Michael Kelly, David Cornutt, David Bar-Or

**Affiliations:** 1 Penrose Hospital, 2222 N Nevada Ave, Colorado Springs, CO 80907 USA; 2 Injury Outcomes Network (ION) Research, 501 East Hampden Ave, Englewood, CO 80113 USA; 3 Swedish Medical Center, 501 East Hampden Ave, Englewood, CO 80113 USA; 4 St. Anthony Hospital, 11600 W 2nd Plaza, Lakewood, CO 80228 USA; 5 Wesley Medical Center, 550 North Hillside St. Wichita, Wichita, KS 67214 USA; 6 Medical City Plano, 3901 West 15th Street, Plano, TX 75075 USA; 7 Research Medical Center, 2316 East Meyer Blvd, Kansas City, MO 64132 USA; 8 Regional West Medical Center, 4021 Ave B, Scottsbluff, NE 69361 USA

**Keywords:** Direct oral anticoagulant, Reversal, Geriatric trauma, Hip fractures

## Abstract

**Background:**

Reversal of direct oral anticoagulants (DOACs) is currently recommended prior to emergent surgery, such as surgical intervention for traumatic geriatric hip fractures. However, reversal methods are expensive and timely, often delaying surgical intervention, which is a predictor of outcomes. The study objective was to examine the effect of DOAC reversal on blood loss and transfusions among geriatric patients with hip fractures.

**Methods:**

This retrospective propensity-matched study across six level I trauma centers included geriatric patients on DOACs with isolated fragility hip fractures requiring surgical intervention (2014–2017). Outcomes included: intraoperative blood loss, intraoperative pRBCs, and hospital length of stay (HLOS).

**Results:**

After matching there were 62 patients (31 reversed, 31 not reversed), 29 patients were not matched. The only reversal method utilized was passive reversal (waiting ≥ 24 hours for elimination). Passively reversed patients had a longer time to surgery (mean, 43 vs. 18 hours, *p* < 0.01). Most patients (92%) had blood loss (90% passively reversed, 94% not reversed); the median volume of blood loss was 100 mL for both those groups, *p* = 0.97. Thirteen percent had pRBCs transfused (13% passively reversed and 13% not reversed); the median volume of pRBCs transfused was 525 mL for those passively reversed and 314 mL for those not reversed, *p* = 0.52. The mean HLOS was significantly longer for those passively reversed (7 vs. 5 days, *p* = 0.001).

**Conclusions:**

Passive DOAC reversal for geriatric patients with isolated hip fracture requiring surgery may be contributing to delayed surgery and an increased HLOS without having a significant effect on blood loss or transfusions. These data suggest that passive DOAC reversal may not be necessary prior to surgical repair of isolated hip fracture.

## Background

The use of direct oral anticoagulants (DOACs) is increasing due to the quick onset and offset of DOACs, lower rates of major hemorrhage, lack of the need for coagulation monitoring, and the reduction in the risk of fatal bleeding and intracranial hemorrhage when compared to vitamin K antagonists [1–6]. Despite their relatively short half-life, the development of reversal agents for emergent surgery of patients on DOACs continues, as does the practice of reversing DOACs in the setting of trauma or major bleeding [6–9].

There are two drugs approved by the Food and Drug Administration for DOAC reversal: idarucizumab for the reversal of dabigatran and andexanet alfa for the reversal of apixaban and rivaroxaban [7, 8]. Other products such as fresh frozen plasma (FFP), prothrombin complex concentrate (PCC), cryoprecipitate, dialysis and factor VIIa can also be used for DOAC reversal [1, 10–12]. Elective hip surgery patients are advised to discontinue DOAC treatment 5 days before surgery, whereas traumatic hip fracture patients may have surgery delayed allowing for DOAC elimination often referred to as the “wait and watch” reversal method [11–13]. DOAC reversal poses a unique challenge compared to vitamin K antagonists because reversal methods are expensive, often require reconstitution with a short shelf-life, have very specific indications, and are not available at all hospitals [6, 14].

There has been an anecdotal increase of physicians not reversing DOACs prior to traumatic hip fracture surgery who instead are sending patients straight to surgery because of purported benefits of timely surgery on outcomes. There are a lack of studies examining the effects of surgical intervention without DOAC reversal on blood loss and the need for transfusions among geriatric hip fractures. The purpose of this study was to compare outcomes among geriatric patients with traumatic hip fractures who had their DOAC reversed prior to surgery to those who did not.

## Methods

This retrospective propensity-matched study across six US level I trauma centers included geriatric patients, aged ≥ 65 years, admitted from 1/1/2014 to 12/31/2017 with an isolated fragility hip fracture (caused by a fall) requiring surgical intervention recorded in these six centers trauma registries. Patients’ medical records were examined for pre-injury medications, and patients who were not taking pre-injury DOACs were excluded, as were patients who were taking pre-injury antiplatelets or pre-injury therapeutic heparin. All centers institutional review boards reviewed and approved of this study.

Patients who were reversed were matched 1:1 to patients who were not reversed prior to surgery using propensity scores. A 1:1 ratio was used as there were not enough matching patients to use a larger ratio, i.e., 1:2 or 1:3. Propensity scores were created using a stepwise logistic regression model, having a caliper distance of 0.001, an entry criterion of 0.20 and an exit criterion of 0.05. Variables available to the model were significantly different in the univariate analysis before matching which can be seen in Table 1. Variables that stayed in the logistic model included the following: comorbidity count, presence of dementia, or having congestive heart failure.

**Table 1:** Patient demographic and clinical characteristics

	Prior to matching (*n* = 91)			Matched (*n *= 62)		
	Not reversed (*n* = 40)	Reversed (*n* = 51)	*P*	Not Reversed (*N* = 31)	Reversed (*N* = 31)	*P*
Age*	81 (8)	82 (8)	** < 0.01**	81 (8)	81 (8)	0.96
Sex, % female (*n*)	30% (12)	47% (24)	0.10	65% (20)	81% (25)	0.33
Race, % white (*n*)	98% (39)	100% (51)	0.44	97% (30)	100% (31)	> 0.99
Prior fracture	3% (1)	16% (8)	0.07	3% (1)	13% (4)	0.25
Walking independent preinjury, % (*n*)	63% (25)	55% (28)	0.39	58% (18)	45% (14)	0.31
Comorbidities						
CHF	10% (4)	29% (15)	**0.02**	13% (4)	13% (4)	> 0.99
Dementia	5% (2)	22% (11)	**0.03**	6% (2)	6% (2)	> 0.99
COPD	3% (1)	20% (10)	**0.02**	3% (1)	13% (4)	0.38
Diabetes	13% (5)	27% (14)	0.08	19% (6)	23% (7)	0.73
CVA	3% (1)	14% (7)	0.07	3% (1)	13% (4)	0.38
Hypertension	63% (25)	69% (35)	0.66	68% (21)	65% (20)	> 0.99
CAD	3% (1)	0% (0)	0.44	3% (1)	0% (0)	> 0.99
Smoker	5% (2)	6% (3)	> 0.99	6% (2)	6% (2)	> 0.99
Comorbidity count	2 (1)	3 (2)	** < 0.01**	2 (1)	2 (1)	0.62
Pre-injury medications						
Beta blocker	35% (14)	59% (30)	**0.03**	32% (10)	61% (19)	**0.03**
Analgesic	28% (11)	24% (12)	0.81	19% (6)	23% (7)	> 0.99
Narcotic	23% (9)	27% (14)	0.63	26% (8)	26% (8)	> 0.99
Antibiotic	5% (2)	12% (6)	0.46	3% (1)	6% (2)	> 0.99
Steroid	18% (7)	24% (12)	0.48	19% (6)	26% (8)	0.75
Calcium	13% (5)	8% (4)	0.50	10% (3)	6% (2)	> 0.99
Pre-injury DOAC medication						
Rivaroxaban	65% (26)	47% (24)	0.09	61% (19)	48% (15)	0.48
Apixaban	33% (13)	39% (20)	0.66	35% (11)	42% (13)	0.79
Dabigatran	3% (1)	12% (6)	0.13	3% (1)	6% (2)	> 0.99
Edoxaban	0% (0)	2% (1)	> 0.99	0% (0)	3% (1)	> 0.99
DOAC indication						
Atrial fibrillation	68% (27)	78% (40)	0.34	68% (21)	81% (25)	0.42
CAD	13% (5)	10% (5)	0.74	13% (4)	6% (2)	0.69
DVT or PE	15% (6)	12% (6)	0.76	13% (4)	13% (4)	> 0.99
Stroke or CVA	8% (3)	8% (4)	> 0.99	6% (2)	6% (2)	> 0.99
Fracture type						
Femoral neck	63% (25)	57% (29)	0.57	58% (18)	58% (18)	> 0.99
Intertrochanteric	38% (15)	39% (20)	0.87	42% (13)	39% (12)	> 0.99
Subtrochanteric	0% (0)	4% (2)	0.50	0% (0)	3% (1)	> 0.99
Surgical procedure^a^						
Intramedullary fixation	45% (18)	43% (22)	> 0.99	55% (17)	35% (11)	0.21
Hemiarthroplasty	25% (10)	31% (16)	0.64	26% (8)	32% (10)	0.77
Internal fixation	18% (7)	25% (13)	0.45	13% (4)	29% (9)	0.18
Bipolar hemiarthroplasty	20% (8)	8% (4)	0.09	77% (24)	10% (3)	0.34
Total hip arthroplasty	20% (8)	4% (2)	**0.02**	13% (4)	6% (2)	0.63
Individual screw placement	3% (1)	4% (2)	> 0.99	0% (0)	3% (1)	> 0.99
Pre-operative hemoglobin^a^	13.4 (2.3)	12.0 (3.2)	** < 0.01**	13.5 (2.1)	11.5 (2.8)	0.78
Abnormal CrCl, % (*n*)	88% (35)	100% (51)	**0.01**	87% (27)	100% (31)	> 0.99
Arrival to surgery, hours^a^	18 (11)	43 (21)	** < 0.01**	18 (12)	43 (22)	** < 0.01**
Last dose to surgery, hours^b^	21 (16, 23)	55 (37, 71)	** < 0.01**	20 (15, 23)	55 (37, 71)	** < 0.01**

^a^Mean (SD)

^b^Median (IQR)

^c^Some patients had multiple surgeries

*DOAC* direct oral anticoagulant, *DVT* deep vein thrombosis, *PE* pulmonary embolism, *CVA* cerebrovascular accident, *CAD* coronary artery disease, *CHF* congestive heart failure, *COPD* chronic obstructive pulmonary disease. Bold values indicate statistical significance at p<0.05

Methods considered as DOAC reversal were: idarucizumab, FFP, cryoprecipitate, PCC, factor VIIa, or passive reversal utilizing the “wait and watch” method. Patients who went to surgery more than 24 hours after the last dose of DOAC medication were considered passively reversed using the wait and watch method. The 24 hour cut-off was used based on the DOAC medications half-lives when the bioavailability would be reduced to less than 10%. The decision to reverse was at the discretion of the treating physicians, thus the six participating centers were not following one uniform protocol for DOAC reversal.

Variables that were abstracted from the patients’ medical records or the trauma registry included the following: age, sex, race, prior hip fracture, indication for DOAC medication, comorbidities [congestive heart failure, dementia, chronic obstructive pulmonary disease (COPD), diabetes, cerebrovascular disease, hypertension, coronary artery disease, seizure disorder, smoker, anemia], pre-injury medications (beta-blockers, analgesics, narcotics, antibiotics, steroids, calcium), DOAC type, admission laboratory results [alanine transaminase, potassium, creatinine clearance (reported as % abnormal, defined as less than 60 mg/L), systolic blood pressure (SBP), heart rate, oxygen saturation, temperature, Glasgow Coma Scale], time from arrival to surgery, time from the last dose of DOAC to surgery, type of fracture, type of procedure, reversal methods, early blood loss (within six hours of arrival), early packed red blood cell (pRBC) transfusion (within six hours of arrival), and early FFP transfusion (within six hours of arrival), intraoperative blood loss, intraoperative pRBC transfusion, and intraoperative FFP transfusion.

The primary outcome was the total volume of intraoperative blood loss which included blood collected by drain or estimated blood loss from soaked sponges. All intraoperative blood loss recorded in the medical record was included in the analysis. Secondary outcomes included any intraoperative blood loss (% with volume > 0 mL), total volume of intraoperative pRBC transfusion, receipt of intraoperative pRBC transfusion (% with volume > 0 mL), pre-operative hemoglobin, intensive care unit (ICU) length of stay (LOS), hospital length of stay (HLOS, defined as hospital admission through discharge in days), complications [pulmonary embolism (PE), unplanned return to the ICU, pneumonia, deep vein thrombosis (DVT), unplanned intubation, cardiac arrest with cardiopulmonary resuscitation (CPR), myocardial infarction (MI), stroke or cerebrovascular accident (CVA)], discharge disposition, and in-hospital mortality. Volumes are reported in milliliters (mL).

Statistical analysis was performed with SAS V9.4 (SAS, Cary, North Carolina, USA) and alpha < 0.05. Continuous variables were summarized as mean (standard deviation) and median (interquartile range) when appropriate based on the distribution of the data. Dichotomous and categorical data are summarized as proportion (count). Paired Student’s t-tests, Wilcoxon paired rank sum test and McNemar’s tests were used when appropriate.

## Results

### Overall population

There were 91 patients identified who were taking pre-injury DOACs (rivaroxaban (55%), apixaban (36%), dabigatran (8%), and edoxaban (1%). Of those, 51 were reversed and 40 were not. Across all 91 patients, the mean age was 81, 40% were male and 99% were white. Prior to matching, patients who were reversed were significantly older (*p* < 0.01), were more likely to have congestive heart failure (*p* = 0.02), dementia (*p *= 0.03), COPD (*p* = 0.02), had a higher comorbidity count (*p* < 0.01), and were more likely to be taking beta-blockers (*p* = 0.03) (Table 1).

### Patients who were not matched

Among patients who were not matched (9 not reversed, 20 reversed), there were no differences in the proportion of patients who died in-hospital (*p* > 0.99), received intraoperative pRBCs (*p* > 0.99), or had intraoperative blood loss (*p* > 0.99). Nor were there differences in ICU LOS (*p* = 0.68), the volume of intraoperative blood loss (*p* = 0.14) or volume of intraoperative pRBCs (*p* = 0.65). Time from hospital arrival to surgery (*p* = 0.003), time from the last dose to surgery (*p *< 0.001) and HLOS (*p* = 0.02) were significantly longer among patients reversed. Of the unmatched patients who were reversed, 90% were passively reversed, 5% were reversed with FFP, and 5% were reversed with FFP and the wait and watch method.

### Matched population

**Fig. 1:**
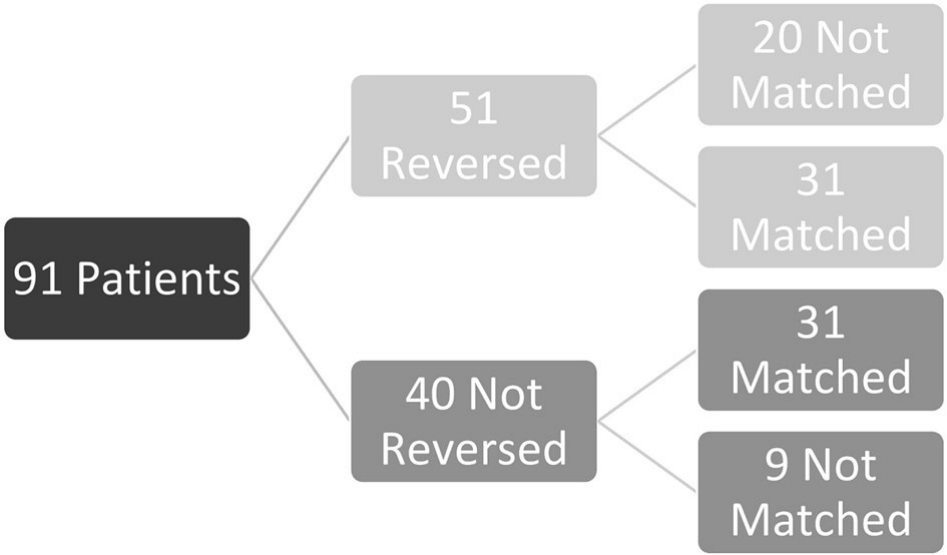
A flowchart describing the enrollment process

Twenty-nine of the 91 patients were excluded after matching. There were 62 patients included in the matched analysis, 31 reversed matched to 31 not reversed, Fig 1. The wait and watch method was the only method of DOAC reversal utilized, therefore we will term the reversed group “passively reversed” for the remainder of the paper. Of those passively reversed, 61% (19) were noted to have surgery delayed more than 24 hours specifically due to concern of the DOAC effect, 23% (7) had delayed surgery for medical clearance, 3% (1) were delayed due to a lack of an operating room, and 13% (4) did not have a reason for a surgical delay of more than 24 hours dictated in the patient’s electronic medical record. Across all matched patients the mean age was 81.5 years old, 44% were male, and 98% were white. Patients were well matched for all matching variables as well as age, sex, race, comorbidities, and admission laboratory results (Table 1). There was still a significantly lower proportion of patients taking beta-blockers pre-injury who were not reversed compared to those passively reversed, 19% vs. 31%, *p* = 0.03. Other pre-injury medications were statistically similar. Across both groups, the most common indication for DOACs was atrial fibrillation (*n* = 46), followed by DVT or PE (n = 8) and CAD (*n* = 6). There were no patients in either group who needed early FFP or pRBC transfusions (within the first six hours of admission); two patients who were not reversed experienced early blood loss, in volumes of 100 mL and 250 mL, while no other patients had early blood loss (within the first six hours of admission).

Compared to those not reversed, the passively reversed patients had a longer mean time to surgery (43 vs. 18 hours, *p* < 0.01) as well as a longer median time from the last dose of DOACs to surgery (55 vs. 20 h, *p* < 0.01). The most common hip fracture type was a fracture of the femoral neck (58%), there were no differences in fracture type. The surgical procedure was also similar between groups. Intramedullary fixation was the most common procedure (45%), followed by hemiarthroplasty (29%). The median pre-operative hemoglobin was also similar between groups, 13.5 vs. 11.5, *p* = 0.78.

Most patients (92%) had intraoperative blood loss (90% passively reversed and 94% not reversed) (Table 2). The median volume of intraoperative blood loss was 100 mL for both those passively reversed and those not reversed, *p* = 0.97. Only 13% of patients had intraoperative pRBCs transfused (13% passively reversed and 13% not reversed). The median volume of pRBCs transfused intraoperatively was 525 mL for those passively reversed and 314 mL for those not reversed, *p* = 0.52. The median HLOS was significantly longer for those passively reversed compared to those not reversed (7.2 vs. 4.7 days, *p* = 0.001). The ICU LOS was not statistically different, p = 0.56. In-hospital complications were rare. Among those passively reversed, 6% (*n* = 2) developed pneumonia, 3% (*n* = 1) had an MI, 3% (n = 1) had an DVT. Among those not reversed, 3% (*n* = 1) patient had an unplanned return to the ICU and 3% (*n* = 1) had an MI. There were no patients with unplanned intubation, cardiac arrest requiring CPR, pulmonary embolism, stroke or CVA. There was a lower proportion of patients who were not reversed and were discharged to rehabilitation (16% vs. 42%, *p* = 0.01). There were no patients who died in-hospital.

**Table 2:** Complications and outcomes

	Not reversed (*N* = 31)	Reversed (*N* = 31)	*P*
Total intraoperative blood loss volume (mL), median (IQR)	100 (35, 150)	100 (25, 200)	0.97
Any intraoperative blood loss, % (*n*)	94% (29)	90% (28)	> 0.99
Total intraoperative pRBC volume (mL), mean (SD)	313.8 (21.4)	525.0 (202.1)	0.52
Receipt of intraoperative pRBCs, % (*n*)	13% (4)	13% (4)	> 0.99
HLOS, mean (SD)	4.7 (2.1)	7.2 (3.0)	**0.001**
ICU LOS, mean (SD)	2.8 (0.8)	4.4 (3.2)	0.56
Death	0% (0)	3% (1)	> 0.99
Complications			
Pneumonia	0% (0)	6% (2)	> 0.99
Unplanned return to ICU	3% (1)	0% (0)	> 0.99
MI	3% (1)	3% (1)	> 0.99
DVT	0% (0)	3% (1)	> 0.99
Discharge disposition^a^			
Home or home with health services	23% (7)	16% (5)	0.75
Rehabilitation	16% (5)	42% (13)	**0.01**
Skilled nursing facility	58% (18)	35% (11)	0.09

*pRBC* packed red blood cells, *HLOS* hospital length of stay, *ICU LOS* intensive care unit length of stay, *ICU* intensive care unit, *MI* myocardial infarction, *DVT* deep vein thrombosis. Bold values indicate statistical significance at p<0.05

^a^Other discharge dispositions hospice (one reversed) and left against medical advice (one not reversed) were not significantly different

### Examining the effect of beta-blocker use on blood loss

There was no difference in the proportion of patients with blood loss when compared by pre-injury beta-blocker use (76% of those not on pre-injury beta-blockers vs. 83% of those on beta-blockers, *p* = 0.50); nor was there a difference in the median (IQR) volume of intraoperative blood loss [100 mL (30, 150) for those not on pre-injury beta-blockers vs. 100 mL (50, 175) for those on pre-injury beta-blockers, *p* = 0.49]. In a stratified analysis of patients on pre-injury beta-blockers, there was no difference in the proportion of patients with blood loss by reversal status (80% not reversed vs. 84% reversed, *p* = 0.79); nor was there a difference in the median (IQR) volume of blood loss [100 mL (75, 125) among those not reversed vs. 75 mL (50, 200) among those reversed, *p* = 0.76]. Among only patients who were not taking pre-injury beta-blockers, the proportion of patients with blood loss was still similar (81% not reversed vs. 67% reversed, *p* = 0.42), as was the median (IQR) volume of blood loss [75 mL (35, 150) among those not reversed vs. 100 mL (25, 175) among those reversed, *p* = 0.76].

## Discussion

This propensity matched analysis demonstrated that the rates and volumes of blood loss and transfusion among geriatric patients with traumatic hip fractures were similar among those passively reversed and those not reversed. Passive DOAC reversal was associated with delayed surgery and longer HLOS, which could increase overall cost to the patient and to the hospital to treat these patients [15]. To our knowledge, this is the first study examining how passive reversal effects volumes of blood loss and transfusion among this population; however, blood transfusions in this population were rare. Previous studies have examined the effect of reversing of both vitamin K antagonists and DOACs, vitamin K antagonists alone, or have compared outcomes among patients taking DOACs to those who were not. [16–22] While many literature reviews and guidance documents have been created on anticoagulant reversal, there is a lack of clinical data specifically on DOAC reversal for geriatric hip fractures [1, 3, 5, 8, 9, 23–34]. With the increasing use of DOACs over vitamin K antagonists, it is important to examine the effects of DOAC reversal separate from vitamin K antagonist reversal.

Current guidance from the American Academy of Orthopaedic Surgeons recommends surgery within 48 hours of arrival and to delay surgery for patients who were taking antiplatelets, but they have no guidelines on surgical delays for patients who were taking anticoagulants, or moreover specifically for DOAC reversal [35]. However, they do state that the presence of comorbidities has a confounding effect on the effect of surgical timing on outcomes and suggest that this subset of patients who have comorbidities could benefit from earlier surgery [35]. The National Institute for Health and Care Excellence (NICE) recommends surgery on the day of or the day after admission with corrective treatment of comorbidities including anticoagulation, but does not not provide specific details on the corrective treatment [36]. The Eastern Association for the Surgery of Trauma (EAST) also states that ideally there would be reduction of all modifiable risk factors and optimization of patients prior to surgery and anesthesia, but also does not mention DOACs specifically, or the reversal of them [37, 38].

A common reason to reverse DOACs before surgery is the risk for bleeding [1–6]. Alternatively, delaying surgery (for reversal) is associated with increased mortality [35, 37, 38]. Moia et Squizzato summarized current reversal strategies and concluded that DOAC reversal should only occur in the presence of DOAC-associated major or life-threatening bleeding, trauma, emergency surgery, or invasive procedures [39]. By their definition, all the patients in this study who suffered traumatic hip fractures should be reversed; however, across all patients only 56% (51/91) were reversed. It was thought among our physicians that patients with DOAC reversal may have been based on patients presenting with blood loss; however, among the passively reversed patients included, there were no patients with pre-operative blood loss. The only patients who had pre-operative blood loss, were those who were not reversed. Moia et Squizzato also stated that the annual rate of anticoagulant-associated bleeding is between 1.5 and 3.5%, but there are a lack of studies with a suitable control arm showing how reversal impacts bleeding highlighting the need for a study such as this [39]. Recently, Shah et al. published a literature review on DOAC reversal for hip fractures and suggest to clearly document the timing of the last dose of DOAC on arrival and conduct a coagulation profile with full blood count along with renal and liver profile [40]. They state to use the creatinine clearance (CrCl) as a marker of safety for surgery (less than 50 mg/L indicating severe impairment) and to guide the delay of surgery for DOAC dissipation [40]. In the present study, there were more patients who were reversed and had an abnormal CrCl, in line with Shah et al.’s recommendation to reverse patients with renal impairment.

Although we do not know what bleeding was DOAC-associated or injury associated, almost all patients experienced intraoperative blood loss. Estimated blood loss was included in the volume of blood loss and is inherently subjective to the treating physicians estimate, but we found no variance between centers indicating that blood loss estimates were similar between centers. There was also no significant difference in the volume of blood loss between groups, which was low being a median of ~ 100 mL overall patients. Similar to this study, Yoo et al. also reported a statistically similar rate of blood loss [16]. Although not significant, there was a lower volume of intraoperative pRBC transfusions among patients who were not reversed. While passive reversal was not statistically associated with intraoperative blood loss or intraoperative pRBC transfusions, there was a significantly longer HLOS among patients reversed.

It was expected that the HLOS would be longer, given the wait and watch method was defined as surgery more than 24 hours after the last dose of DOACs, but the difference in HLOS was longer than the length of the wait and watch reversal method, two days vs. 24 h. Yoo et al. also reported that patients who were reversed prior to surgery experienced a longer HLOS, but the difference was no longer significant after adjustment [16]. A previous study found that delaying surgery more than 24 hours has shown to increase the time in rehabilitation and HLOS [41]. There was an increase in passively reversed patients who were discharged to rehabilitation in this study, which could also increase overall cost to the patient. It is possible the increased time to surgery, and not the reversal of DOACs, was driving the increased HLOS.

To note, within the matched population there remained a significantly higher proportion of passively reversed patients on pre-injury beta-blockers. Pre-operative beta-blocker use may reduce intraoperative bleeding, prior studies have discussed this could be due to the anti-hypertensive properties of beta-blockers leading to hemodynamic stability [42, 43]. Since there was a higher proportion of reversed patients on pre-injury beta-blockers this could indicate their average blood loss was lower than it would have been if the groups were balanced in their use of  pre-injury beta-blockers. However, when blood loss was examined by pre-injury beta-blocker use, we identified no difference in the rate nor volume of blood loss between groups. Thus, since pre-injury beta-blocker use was not significantly associated with blood loss, it is likely not a confounding the results seen in this study. Additionally in a stratified analysis by pre-injury beta-blocker use, blood loss was still not impacted by reversal status for patients on pre-injury beta-blockers and those who were not on pre-injury beta-blockers. It is possible the pre-injury beta-blocker’s bioavailability has been eliminated enough by the time of surgery that it is not impacting intraoperative blood loss.

While there are tests to indicate the presence of DOACs, they generally do not provide an accurate assessment of the drug’s bioavailability [44, 45]. Specific coagulation assays can be used for the quantification of DOAC plasma levels, but their reliability is questionable [44, 45]. Elevated prothrombin time can indicate the presence of FXa inhibitors (rivaroxaban, apixaban, and edoxaban) but is not accurate for apixaban and edoxaban [44]. Activated partial thromboplastic time, ecarin clotting time, chromogenic ecarin assay or dilute thrombin time can detect dabigatran [44]. But these tests can be affected by factors other than the DOAC [13]. Because of this there remains no way to indicate a safe threshold for surgery when using the wait and watch reversal method [44].

## Limitations

This was a retrospective study with a relatively small sample size and blood transfusions were rare. It was a multicenter study conducted across six level 1 trauma centers over 4 years so may be more generalizable than other previous single center studies. The participating sites were not following standardized reversal protocols or blood transfusion protocols which was conducted at physician’s discretion. The reason why the physician chose to reverse to use was not recorded for 13% (*n* = 4) of the patients who were reversed. The wait and watch method may have been preferred to pharmacological reversal (idarucizumab, FFP, PCC, etc.) due to cost or convenience. There were no methods of pharmacological reversal utilized in this study, only the wait and watch method which may not have been intentional. We defined “wait and watch” based on the time each DOAC mediation is dissipated to less than 10% using the half-life for each medication in a geriatric population. Larger prospective studies are needed to confirm the results. Further research identifying geriatric patients with hip fractures on pre-injury DOACs who did benefit from reversal may be useful.

## Conclusions

Passive DOAC reversal for geriatric patients with isolated hip fractures may be contributing to delayed surgery, an increased HLOS, and an increase in patients discharged to rehabilitation rather than home without having a significant effect on intraoperative blood loss or transfusions. This information may be useful to physicians who are wondering which is more important, timely surgery or delaying surgery for DOAC reversal, when treating geriatric patients with traumatic hip fractures on DOACs.

## Data Availability

The datasets generated and/or analyzed during the current study are not publicly available due to data use agreements with the participating centers and lack of approval from the IRB to transmit data to external sources. Coding used for the analysis can be made upon reasonable request to DBO.

## Abbreviations

DOAC: Direct oral anticoagulant
FFP: Fresh frozen plasma
pRBC: Packed red blood cells
mL: Milliliters
HLOS: Hospital length of stay
PCC: Prothrombin complex concentrate
IRB: Institutional review board
No.: Number
COPD: Chronic obstructive pulmonary disease
ICU LOS: Intensive care unit length of stay
PE: Pulmonary embolism
ICU: Intensive care unit
DVT: Deep vein thrombosis
CPR: Cardiopulmonary resuscitation
MI: Myocardial infarction
CVA: Cerebrovascular accident
CHF: Congestive heart failure
